# Notch ligands regulate the muscle stem-like state ex vivo but are not sufficient for retaining regenerative capacity

**DOI:** 10.1371/journal.pone.0177516

**Published:** 2017-05-12

**Authors:** Hiroshi Sakai, Sumiaki Fukuda, Miki Nakamura, Akiyoshi Uezumi, Yu-taro Noguchi, Takahiko Sato, Mitsuhiro Morita, Harumoto Yamada, Kunihiro Tsuchida, Shahragim Tajbakhsh, So-ichiro Fukada

**Affiliations:** 1 Stem Cells & Development, Department of Developmental & Stem Cell Biology, CNRS UMR 3738, Institut Pasteur, Paris, France; 2 Laboratory of Molecular and Cellular Physiology, Graduate School of Pharmaceutical Sciences, Osaka University, Osaka, Japan; 3 Division for Therapies Against Intractable Diseases, Institute for Comprehensive Medical Science, Fujita Health University, Aichi, Japan; 4 Department of Ophthalmology, Kyoto Prefectural University of Medicine, Kyoto, Japan; 5 Department of Orthopaedic Surgery, Fujita Health University, Aichi, Japan; University of Minnesota Medical Center, UNITED STATES

## Abstract

Myogenic stem cells are a promising avenue for the treatment of muscular disorders. Freshly isolated muscle stem cells have a remarkable engraftment ability in vivo, but their cell number is limited. Current conventional culture conditions do not allow muscle stem cells to expand in vitro with their bona fide engraftment efficiency, requiring the improvement of culture procedures for achieving successful cell-therapy for muscle disorders. Here we expanded mouse muscle stem cells and human myoblasts with Notch ligands, DLL1, DLL4, and JAG1 to activate Notch signaling in vitro and to investigate whether these cells could retain their engraftment efficiency. Notch signaling promotes the expansion of Pax7+MyoD- mouse muscle stem-like cells and inhibits differentiation even after passage in vitro. Treatment with Notch ligands induced the Notch target genes and generated PAX7+MYOD- stem-like cells from human myoblasts previously cultured on conventional culture plates. However, cells treated with Notch ligands exhibit a stem cell-like state in culture, yet their regenerative ability was less than that of freshly isolated cells in vivo and was comparable to that of the control. These unexpected findings suggest that artificial maintenance of Notch signaling alone is insufficient for improving regenerative capacity of mouse and human donor-muscle cells and suggest that combinatorial events are critical to achieve muscle stem cell and myoblast engraftment potential.

## Introduction

Skeletal muscle regeneration has an absolute requirement for muscle stem (satellite) cells [1–3]. Muscle stem cells are mitotically quiescent during homeostasis in the adult mouse, but after their activation, they enter into the cell cycle and proliferate to generate myoblasts. Myoblasts then fuse as myogenic commitment proceeds to make new myofibers. Therefore, myoblast-transfer therapy has been considered to be a promising therapeutic approach for the treatment of muscular disorders, particularly for muscular dystrophies. In early 1990’s, myoblasts were transplanted into patients with Duchenne muscular dystrophy (DMD), but the results of clinical trials were unsuccessful [4–6]. There are some causative factors such as insufficient immune suppression, low survival of donor cells, and the quality of donor cells that can explain these failures. To improve this efficiency, the *bona fide* potential of muscle stem cells was reexamined [7, 8]. Among them, one of the outstanding observations was the comparison of the in vivo regenerative ability between freshly isolated murine muscle stem cells (quiescent satellite cells) and cultured primary myoblasts. Notably, the expansion of muscle stem cells resulted in a dramatic reduction in their regenerative capacity following transplantation [9, 10]. Since the number of freshly isolated muscle stem cells is limited for effective use as a source of donor cells, their in vitro expansion has been considered to be an essential step for achieving successful myoblast transfer therapy. Hence, it is critical to establish the appropriate culture conditions that allow muscle stem cells to expand while maintaining their original engraftment potential.

Quiescent muscle stem cells do not express MyoD protein, however they do so following their activation. During the generation of adult satellite cells following muscle injury, they express MyoD transiently [11], and this expression is likely necessary for their regenerative potential [12]. We showed previously that fetal myogenic progenitors (FMP) can be divided into MyoD+ and MyoD- populations, and MyoD+ FMP have a superior regenerative potential compared to MyoD- FMP [12]. Furthermore, we also compared the regenerative potential of neonatal and adult muscle stem cells, and found that adult muscle stem cells are superior to fetal counterparts [12]. These results suggest that both MyoD-priming and sequential MyoD suppression are necessary for muscle stem cells to acquire robust regenerative ability during development.

The roles of canonical Notch signaling and the effector genes in the suppression of myogenic differentiation, including the inhibition of *MyoD* expression, are well studied [13–17]. In addition, one study reported that one of the Notch ligands, DLL1, improved the efficiency of canine myoblast transplantation in immunodeficient mice [18]. Dll1 is widely used for the induction of Notch signaling in murine myogenic cells [15, 17, 19, 20]. However, the suitable NOTCH ligand for human myogenic cells has not been demonstrated. Furthermore, it is unclear whether the NOTCH ligand can suppress MYOD expression in human myoblasts. Here, we investigated the effect of several NOTCH ligands on the properties of mouse and human myogenic cells in vitro and subsequently examined the transplantation efficiency of the cells treated with NOTCH ligands.

## Results

### NOTCH ligand-treatment alters gene expression in mouse myogenic cells

**Fig 1:**
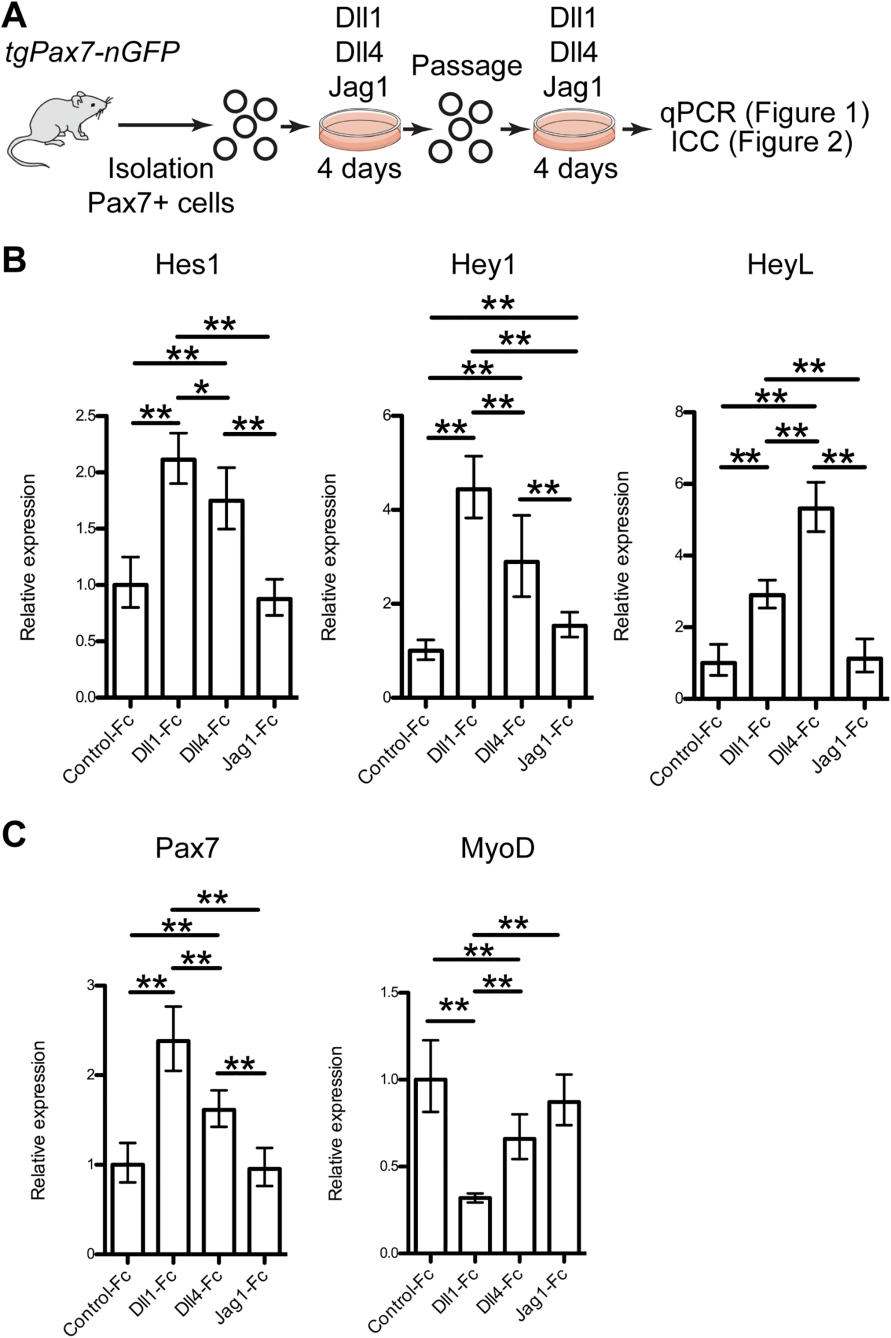
Dll1-Fc, Dll4-Fc, and Jag1-Fc alter gene expression in mouse myogenic cells. (A) Scheme for analysis of Notch ligands-treated mouse muscle stem cells. (B, C) Relative mRNA expression of Notch effector genes (B) and myogenic genes (C) in mouse myogenic cells cultured with Notch ligand (Control-Fc, Dll1-Fc, Dll4-Fc, and Jag1-Fc: 5 ng/μl). Graphs represent average and 95% confidence interval from 3–4 independent experiments. Welch’s t-test was applied with Bonferroni correction. P-values are <0.05 (*) or <0.01 (**).

To determine which Notch ligands can induce endogenous Notch activity and anti-myogenic effects in mice, skeletal muscle stem cells were plated on dishes coated with Notch ligands fused with the Fc domain of human IgG, designated as Dll1-Fc, Dll4-Fc [21], and Jag1-Fc. Muscle stem cells were isolated by fluorescence-activated cell sorting (FACS) using *Tg*:*Pax7-nGFP* mice [22] and then expanded for 4 days on the coated dishes. The regenerative efficiency of cells expanded in vitro without passaging in standard culture conditions was comparable to that of freshly isolated cells, however, passaged cells were reported to lose their engraftment potential [9, 10]. To achieve cell therapy for muscular diseases, maintaining *bona fide* muscle stem cell function following expansion in culture is critical for preparing the large number of cells often required for transplantations. To address this issue, the expanded cells were isolated by FACS using GFP fluorescence then cultured again on coated dishes for an additional 4-days (total for 8 days, Fig 1A). Dll1-Fc and Dll4-Fc induced high levels of expression of *Hey1* and *HeyL* even after 8 days of culture but *Hes1* was activated to a lesser extent (Fig 1B) as we showed previously [13]. Dll1-Fc and Dll4-Fc inhibited *MyoD* expression, whereas *Pax7* mRNA expression was increased slightly (Fig 1C). In contrast, Jag1-Fc induced only *Hey1*, and there was no difference between Jag1-Fc and control-Fc treated cells in *Hes1*, *HeyL*, *MyoD*, or *Pax7* expression (Fig 1B and 1C). Therefore, Dll1-Fc and Dll4-Fc induced Notch signaling and inhibited myogenic differentiation in vitro whereas Jag1-Fc did not promote robust Notch signaling.

### NOTCH ligand-treatment retains *Pax7* expression but represses commitment markers in mouse myogenic cells

**Fig 2:**
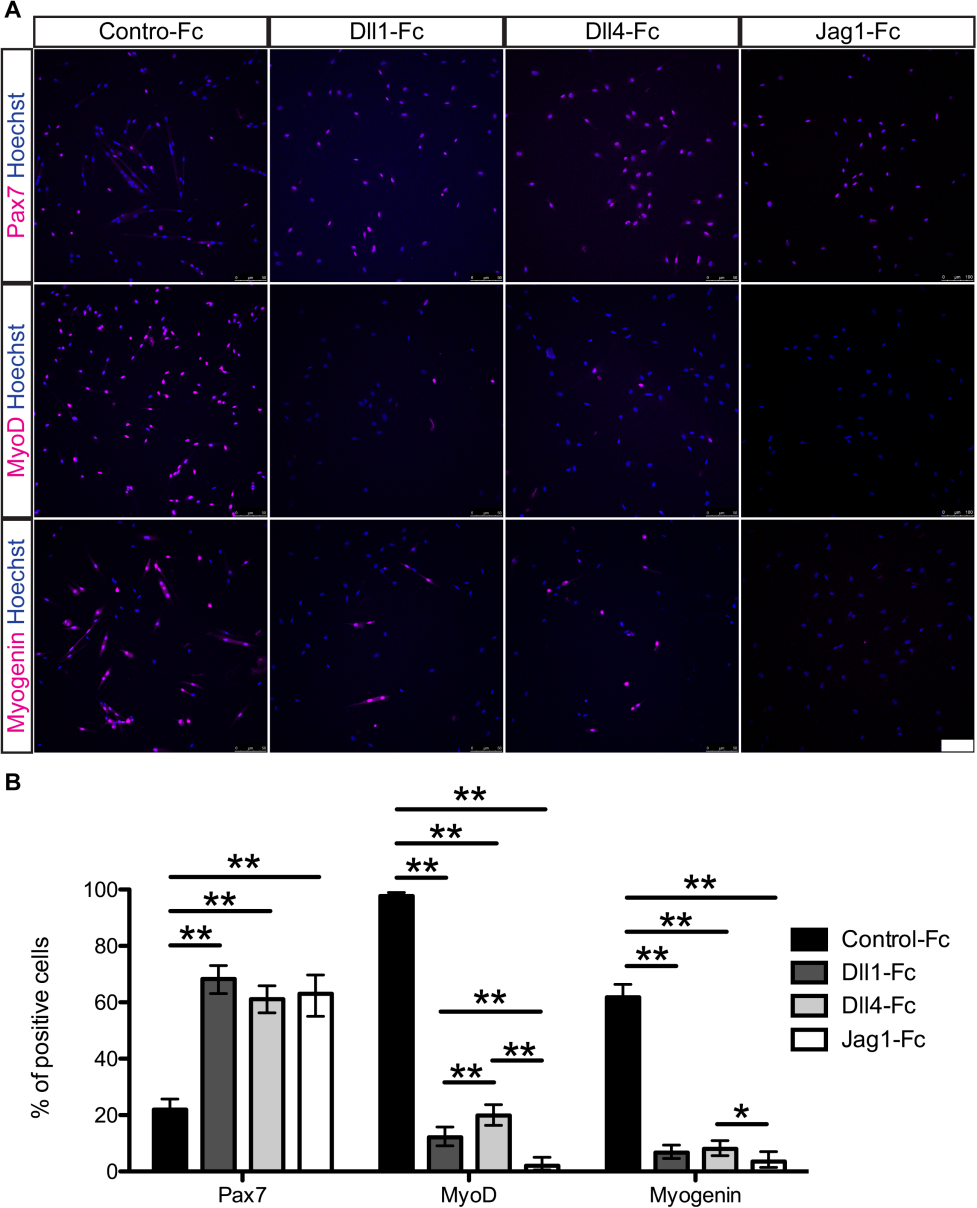
Dll1-Fc, Dll4-Fc, and Jag1-Fc retain Pax7 expression but repress activation markers in mouse myogenic cells ex vivo. (A) Immunocytochemistry for Pax7, MyoD, and Myogenin of mouse myogenic cells cultured with or without Notch ligands. Hoechst staining reveals nuclei. Scale bar = 100 μm. (B) Quantitative analysis of Pax7, MyoD, and Myogenin in the cultured cells. Data are reported as mean and 95% confidence interval of 200–450 cells per staining condition. Statistical significance was assessed by 2-sample test for equality of proportions with Holm method. P-value are <0.05 (*) or <0.01 (**).

To confirm the RT-qPCR results, mouse myogenic cells cultured on Notch ligands were immunostained for Pax7, MyoD, and Myogenin protein after 8 days in culture (Fig 2A). The cells treated with Dll1-Fc, Dll4-Fc, or Jag1-Fc had a similar expression ratio for Pax7, MyoD, and Myogenin (Fig 2B). Myogenic cells in control lost their Pax7 expression (22%) in culture but Dll1-Fc, Dll4-Fc, and Jag1-Fc treatments maintained elevated Pax7 expression levels (68%, 61%, and 63% respectively). MyoD protein was detected in almost all cells in control, whereas this expression was dramatically reduced to 12% for Dll1-Fc-treated and 19% for Dll4-Fc-treated cells. Jag1-Fc strongly suppressed MyoD at the protein level (2%) although there was no difference in transcript levels. For Myogenin expression, 61% of the cells in control were positive for this marker respectively compared with 6.6%, 8%, and 3.5% of the treated cells. These observations suggest that Notch signaling promotes the expansion of Pax7+MyoD- muscle stem-like cells and inhibits differentiation even after passage in vitro.

### Dll1/4-treatment does not enhance engraftment efficiency of mouse donor cells

**Fig 3:**
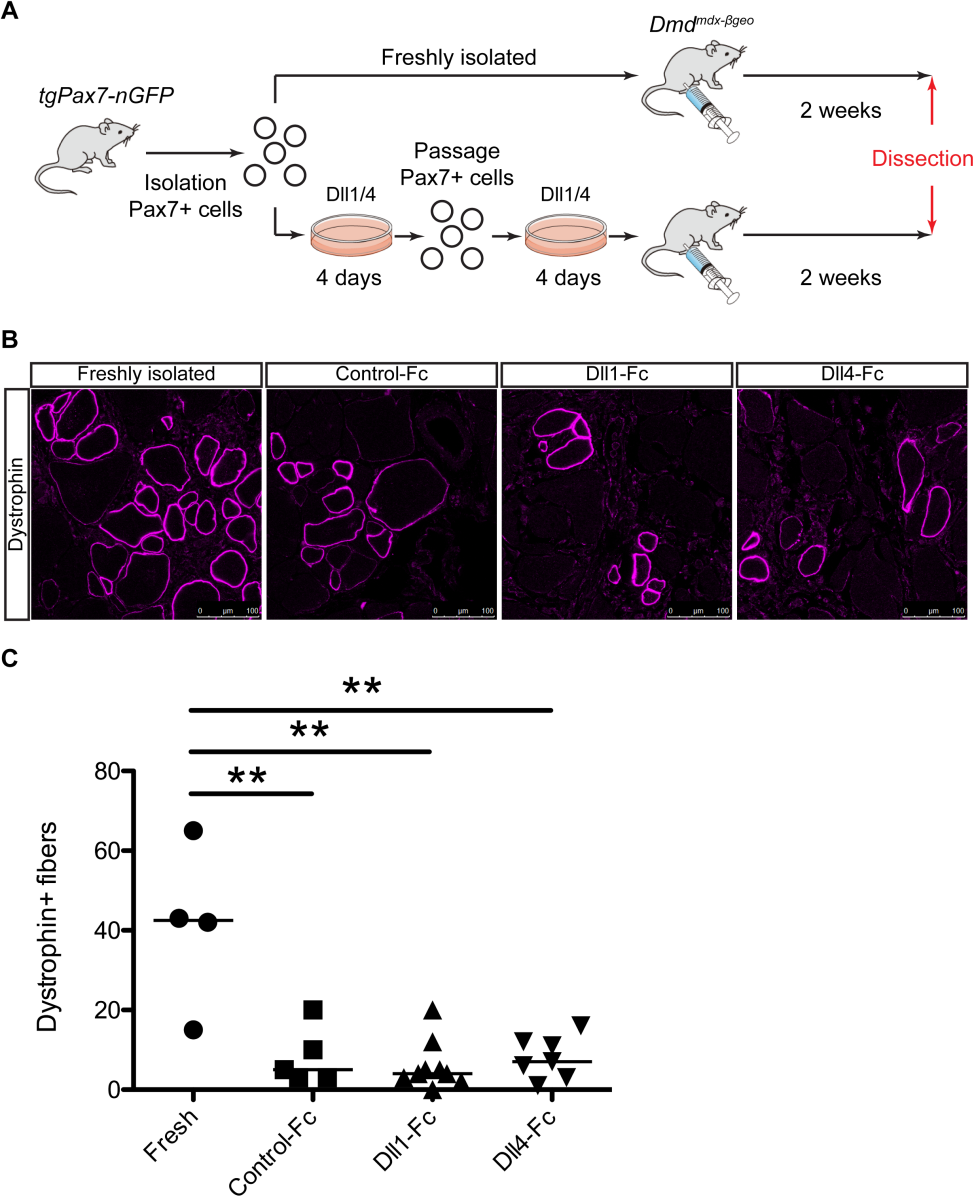
Dll1/4-treatment is not sufficient for retaining regenerative capacity of mouse donor-cells following transplantation. (A) Experimental procedure for the engraftment of NOTCH ligands-treated mouse myogenic cells. (B) Immunostaining for dystrophin in TA muscles of *Dmd*^*mdx-βgeo*^ mice injected with freshly isolated, control-Fc-treated, Dll1-Fc-treated, or Dll4-Fc-treated mouse myogenic cells 2 weeks after intramuscular engraftment. Scale bar = 100 μm. (C) Quantification of dystrophin+ fibers in TA muscle engrafted with freshly isolated (n = 4), control ligand-treated (n = 5), Dll1 ligand-treated (n = 9), and Dll4 ligand-treated cells (n = 7). All data were plotted with the mean from all engrafted mice. Welch’s t-test was applied with Bonferroni correction. P-values are <0.01 (**).

As shown in Fig 2, Jag1-Fc suppressed MyoD protein levels as well as Dll1/4, however, not all Notch downstream targets were effectively induced by Jag1-Fc. In a previous study using canine myogenic cells, both *Hey1* and *HeyL* were induced by DLL1. Therefore, to perform mouse engraftment experiments under similar Notch signaling conditions, we used Dll1-Fc and Dll4-Fc which also induced *Hey1* and *HeyL* in mouse myogenic cells. To assess the transplantation efficiency, 2 x 10^4^ of Pax7-nGFP+ myogenic cells treated with control-Fc, Dll1-Fc, and Dll4-Fc were injected into cardiotoxin-injured *Tibialis anterior* (TA) muscles of dystrophin knock-out mice (*Dmd*^*mdx-βgeo*^ mice) (Fig 3A) [23]. Freshly isolated muscle stem cells were injected as a positive control for the transplantation. The expression of dystrophin in host muscle fibers was evaluated two weeks after engraftment. Immunostaining showed the presence of dystrophin+ fibers in host muscle sections from *Dmd*^*mdx-βgeo*^ mice engrafted with the wild-type myogenic cells (Fig 3B). Quantification of dystrophin+ fibers in transplanted *Dmd*^*mdx-βgeo*^ mice showed that freshly isolated muscle stem cells had the highest efficiency of engraftment (ranging from 15 to 65 fibers) (Fig 3C). The number of dystrophin+ fibres in the analysed muscles was comparable for control-, Dll1-, and Dll4-treated conditions (ranging from 3 to 20 fibers with control myogenic cells, 0 to 20 fibers with Dll1-tretaed cells, and 1 to 16 fibers with Dll4-treated cells, Fig 3C). Therefore, these results show that Dll1/4-treatment in vitro is not sufficient to maintain engraftment efficiency of mouse donor cells.

### The effect of NOTCH ligand on myogenic genes

**Fig 4:**
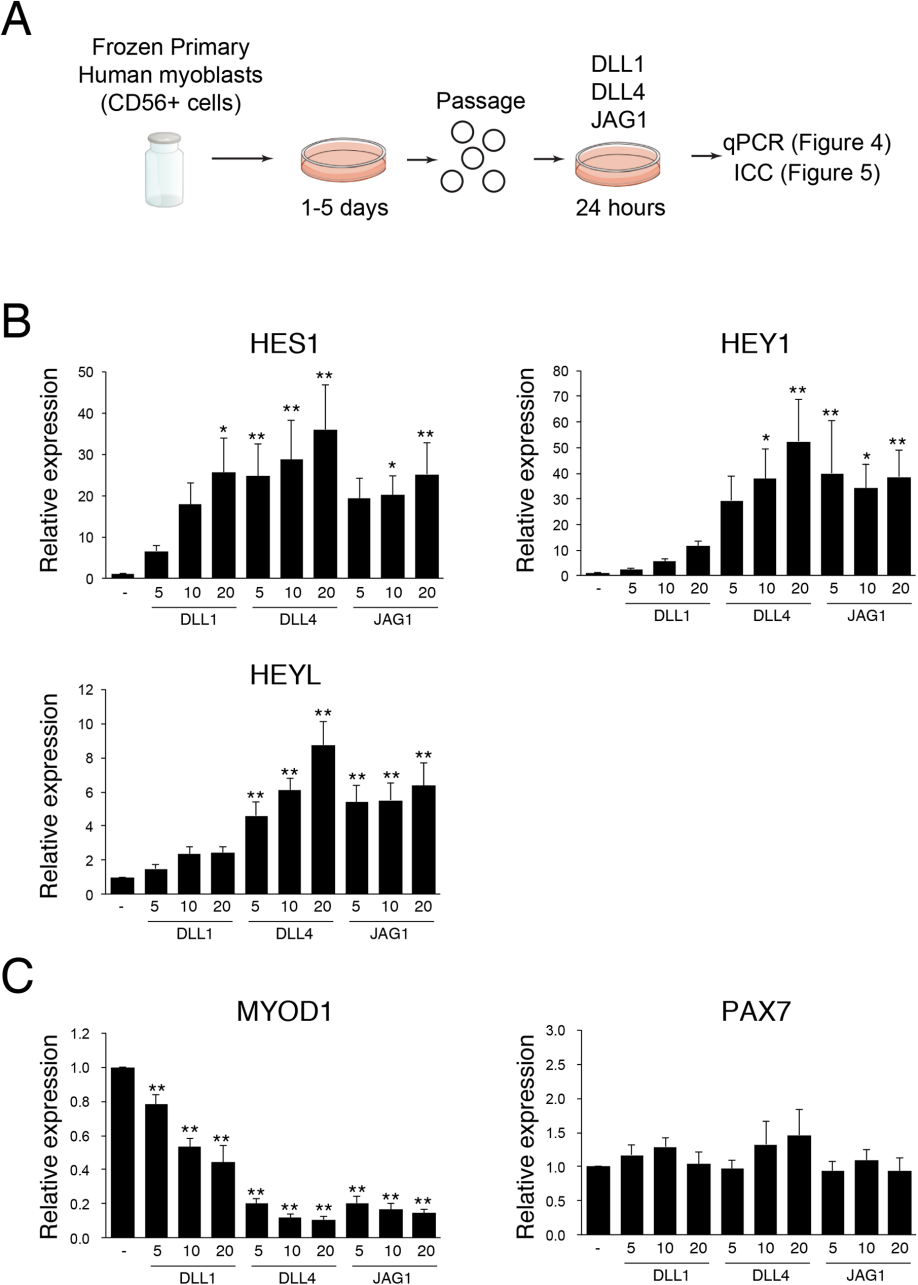
JAG1 and DLL4 significantly suppress *MYOD* mRNA levels in human myoblasts. (A) Scheme for analysis of NOTCH ligands-treated human myoblasts. (B) Relative mRNA expression of NOTCH effector genes in human myoblasts cultured with or without indicated NOTCH ligand (DLL1: 5–20 ng/μl, DLL4: 5–20 ng/μl, JAG1: 5–20 ng/μl). Values are means ± S.D. (n = 3–5). (C) Relative mRNA expression of myogenic genes in human myoblasts cultured with or without the indicated NOTCH ligand. Values are means ± S.D. (n = 3–5). Statistical significance was assessed by non-repeated measures analysis of variance (ANOVA) followed by the Bonferroni test (vs control). P-values are <0.05 (*) or <0.01 (**).

Recently, we reported a purification method for human myoblasts from crude mononuclear cells derived from human muscles using CD56 (NCAM) [24]. As with murine primary myogenic cells, they undergo myogenic differentiation, but exhibit a lesser tendency to assume other cell-fates [25]. In the present study, all human myoblasts were prepared by the same procedure, and were expanded on traditional culture dishes, and frozen at least once before using the following experiments to avoid excessive passage that might compromise engraftment potential (Fig 4A).

Notch signaling strongly induces *Hey1* and *HeyL* mRNA expression in murine myogenic cells after 24 hours of Notch-ligand treatment [17]. Although a negative autoregulatory feedback loop by Hes1 protein obscures the detection of *Hes1* mRNA expression in mouse stem cells, *HES1* mRNA is significantly induced by DLL1 in human myoblasts [26]. To determine the role of NOTCH in human myoblasts, we stimulated human myoblasts with each NOTCH ligand for 24 hours and then examined NOTCH target gene expression. As shown in Fig 4B, DLL4 and JAG1 strongly induced *HES1*, *HEY1* and *HEYL* mRNA expression in human myoblasts, suggesting that these genes were primary targets of NOTCH singling in human myoblasts, as well as murine myoblasts. In contrast, DLL1 induced *HES1* to a similar level with DLL4 and JAG1, but the induction level of *HEY1* and *HEYL* were much weaker than those of DLL4 or JAG1.

Next, we examined the level of myogenic commitment following stimulation of NOTCH activity by measuring mRNA expression of *MYOD* in the cells. As shown in Fig 4C, DLL4 and JAG1 significantly inhibit *MYOD* mRNA expression in human myoblasts. Consistent with the results in Fig 4B, the efficacy of DLL1 was much weaker than those of JAG1 or DLL1. In mouse, *Pax7* mRNA increased by a Notch ligand (Fig 1) [25], but *PAX7* mRNA expression was not altered among the cells stimulated with NOTCH ligands and non-stimulated cells in human myoblasts (Fig 4C).

### The effect of NOTCH ligand on MYOD expression

**Fig 5:**
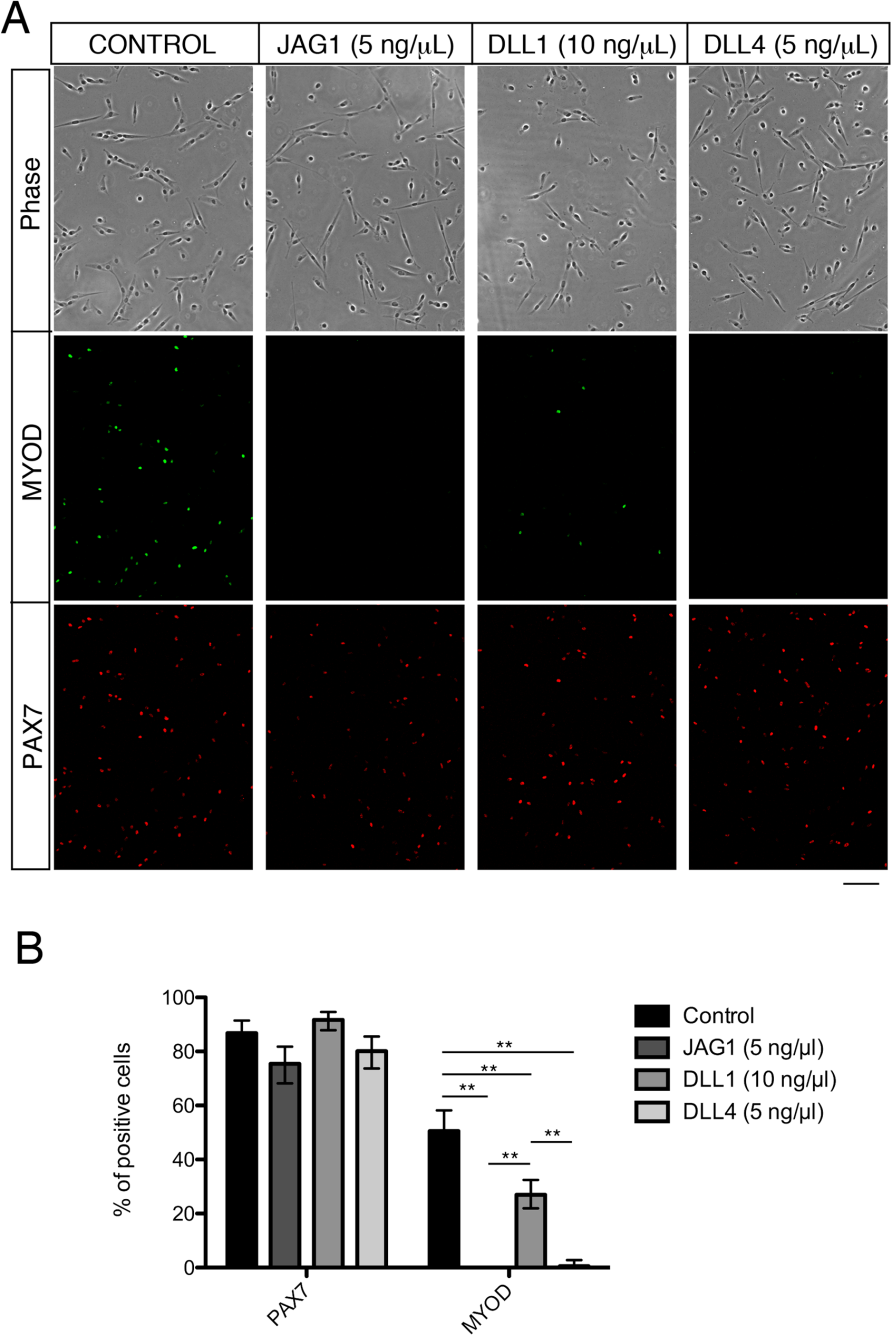
JAG1 and DLL4 significantly suppress MYOD protein level in human myoblasts. (A) Immunocytochemistry of human myoblasts cultured with or without JAG1, DLL1, or DLL4 ligand for PAX7 (red) and MYOD (green). The upper panels indicated the transmission images. Scale bar = 100 μm. (B) Quantitative analyses of PAX7 and MYOD protein expression in human myoblasts cultured with or without NOTCH ligands (JAG1: 5 ng/μl, DLL1: 10 ng/μl, DLL4: 5 ng/μl). Data are reported as mean and 95% confidence interval of 150–200 cells per staining. Statistical significance was assessed by 2-sample test for equality of proportions with Holm method. P-values are <0.01 (**). Representative data are shown, and similar results were obtained using different human myoblasts.

To confirm the results obtained by RT-qPCR, human myoblasts treated with NOTCH ligands were stained with MYOD and PAX7 antibodies. As shown in Fig 5A, JAG1 and DLL4 strongly suppressed MYOD expression at the protein level. In contrast, the effect of DLL1 was modest whereas PAX7 expression was not significantly altered between the cells stimulated with NOTCH ligands and non-stimulated cells (Fig 5B). Based on these observations, we conclude that JAG1 and DLL4 are suitable ligands for inducing PAX7+MYOD- satellite cell-like cells in human myoblasts rather than DLL1.

### In vivo efficacy of NOTCH ligand-stimulated human myoblasts

**Fig 6:**
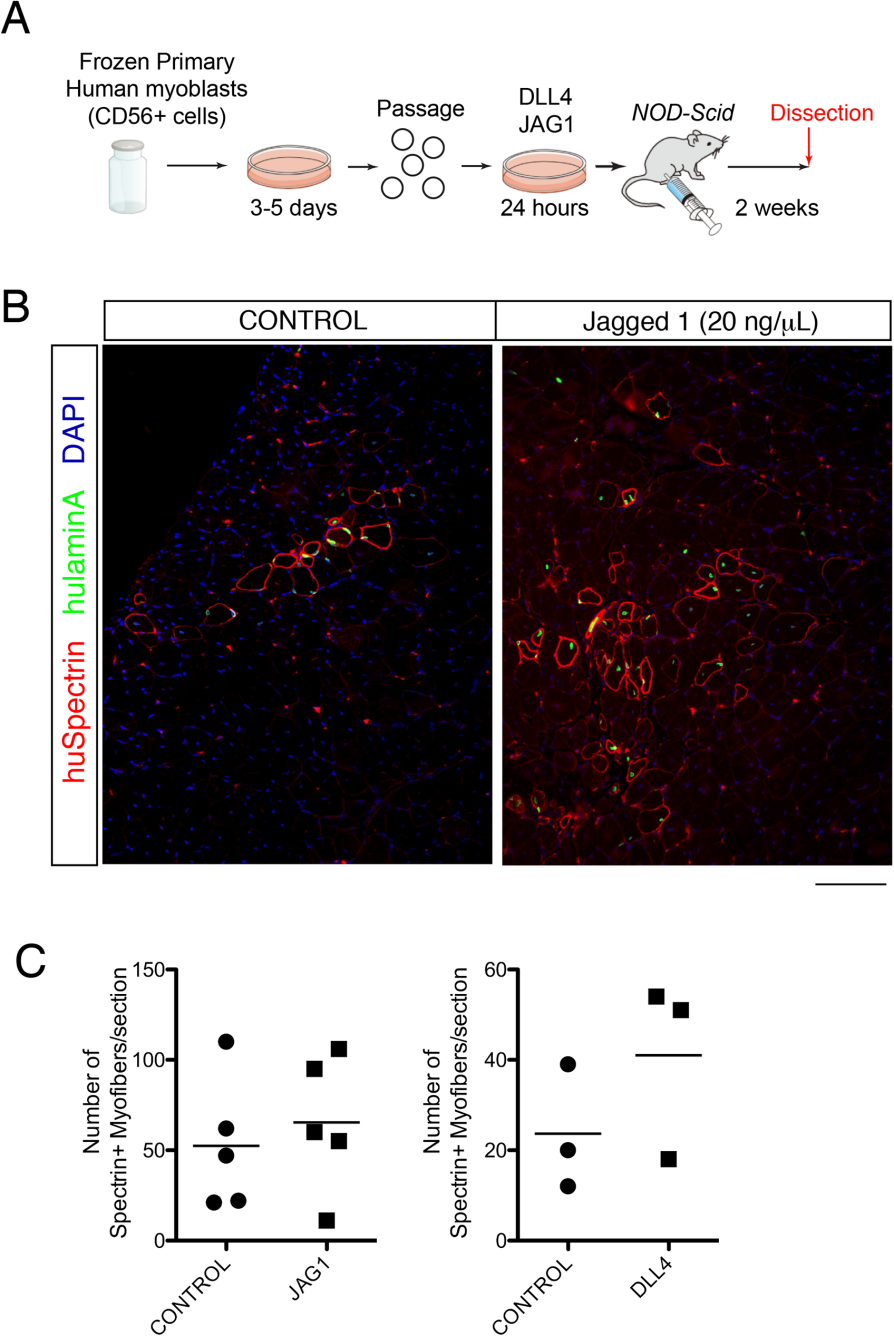
The effect of NOTCH ligands on human myoblast engraftment. (A) Experimental procedure for the engraftment of NOTCH ligands-treated human myoblasts. (B) Immunostaining for human SPECTRIN (red), anti-human LAMIN A/C (green), and DAPI (blue) in TA muscles of NOD-SCID mice engrafted with non- or JAG1 ligand-treated human myoblasts 2 weeks after transplantation. Scale bar = 100 μm. (C) Quantification of SPECTRIN+ fibers in TA engrafted with control- (n = 5, n = 3), JAG1- (n = 5), or DLL4- (n = 3) treated human myoblasts. All data were plotted with the mean from all engrafted mice. Welch’s t-test was applied.

To elucidate the efficacy of NOTCH ligands JAG1 and DLL4 on *in vivo* engraftment of human myoblasts, 1 x 10^5^ cells that were NOTCH ligand-treated or non-treated were transplanted into TA muscle of immunocompromised NOD-SCID mice that received cardiotoxin injection one day before the transplantation, and compared to controls in the contralateral TA muscle (Fig 6A). Two weeks after the transplantation, the engrafted muscles were fixed and the engraftment efficiency was determined by counting human SPECTRIN+ myofibers (Fig 6B). As shown in Fig 6C, the maximum number of SPECTRIN+ fibers in each injected muscle was comparable for control-, JAG1- and DLL4-treated conditions. (ranging from 21–110 fibers with control cells for JAG1, 11–106 fibers with JAG1-treated cells, 12–39 fibers with control cells for DLL4, and 18–54 fibers with DLL4-treated cells). These results suggest that although the NOTCH ligands JAG1 and DLL4 significantly suppress MYOD expression, NOTCH signaling alone is not sufficient to improve the engraftment potential of human myoblasts, as was observed with murine myogenic cell engraftment.

## Discussion

Muscle satellite cells play a crucial role in skeletal muscle regeneration following muscle injuries [1, 2]. As skeletal muscles undergo repeated bouts of injury following trauma or excessive exercise, these muscle stem cells are considered to be essential for sustaining skeletal muscle function throughout life [1]. In the context of disease, muscle stem cells have been explored as a principal cell source for the treatment of these muscular disorders that include muscular dystrophies. However, obtaining sufficient amounts of freshly isolated muscle stem cells for cell therapy in a major challenge, hence the necessity of in vitro expansion of primary myogenic cells that will retain robust engraftment potential. In attempts to address this challenge, one study reported that 12 kPa soft gel (substrate elasticity) improves mouse muscle stem cell-transplantation efficiency [27]. In another study using human myoblasts, p38 inhibitor was reported to improve the efficiency of engraftment of the cultured human muscle stem cells. Interestingly, cells treated with p38 inhibitor showed a higher transplantation efficiency when compared to freshly isolated human muscle stem cells [28]. By RNA-seq analyses, the authors found that genes associated with ageing were downregulated by the p38 inhibitor compared to freshly isolated human muscle stem cells, suggesting the downregulation of those genes resulted in the superior engraftment ability of the treated myoblasts. In the context of Notch signaling, Parker et al. reported that Notch ligand (DLL1) improves the efficacy of canine myoblast transplantation [18], in contrast to our present studies with murine and human myogenic cells. It is not clear if this differential requirement for Notch in the context of transplantations is due to species-specific differences, however, some points need consideration. Between the canine report and our study, there are three principal differences; 1) The present study used purified mouse muscle stem cells or human myoblasts whereas the report by Parker et al. used muscle-derived cells that might have included an undetermined amount of other cell types, as purification by FACS was not included in the protocol. Given that co-transplantation of myoblasts with other cell types can improve engraftment of efficiency [29], canonical NOTCH might indirectly effect the efficacy of myoblast transplantation in a mixed population of cells; 2) The present study used previously cultured human cells on standard tissue culture plates. This difference might explain the discrepancy with the results by Parker and colleagues who reported that DLL1 did not improve the engraftment efficiency of previously culture cells on standard tissue culture plates; 3) Parker et al. cultured freshly isolated muscle-derived cells for 8 days whereas in our study human myoblasts were cultured on NOTCH ligands for 1 day. The difference of culture period might influence the differentiated state of the cells. In this context, it is not clear to what extent NOTCH ligands remain biologically active in culture without replenishment.

Recent studies suggested that JAG1 might act to rescue DMD phenotypes in Golden Retriever muscular dystrophy dogs, as this ligand was elevated in dogs that were mildly affected by the mutation [30]. Genome-wide mapping analyses revealed a single point mutation in the promoter region of *Jag1*, and this was associated with the partial rescue of the DMD phenotype in the dog [31]. That study also showed that in vivo overexpression of Jag1 rescues the muscle phenotype in a dystrophic *sapje* zebrafish DMD model. The mechanism by which JAG1 improves dystrophic phenotypes in dog and fish remains to be elucidated, but it is speculated that JAG1 significantly improves muscle regenerative ability, rather than inhibition of degeneration, since an absence of muscle dystrophin, elevated serum CK levels, and lack of evidence of utrophin upregulation were confirmed. Since in vitro JAG1 treatment did not have a significant impact on the improvement of human myoblast engraftment efficiency, it remains possible that continuous JAG1 stimulation in vivo would be necessary for the improvement of myoblast-engraftment.

Some mechanisms underlying responsiveness of Notch to the ligand need to be considered. For example, the sugar translation enzyme Fringe is a glycosyltransferase that modifies Notch receptors. Unmodified Notch binds to Serrate/Jagged rather than Delta, and glycosylated Notch prefer to bind to Delta [32]. In addition, Yamamoto et al. performed genetic screens and isolated a mutant Drosophila having a missense mutation in epidermal growth factor repeat-8 (EGFr-8) that is defective in Serrate but not in Delta-dependent signaling [33]. In this context, Notch receptor can recognize Delta or Jagged ligand in a structure-based manner, which might explain the different expression patterns of Notch effector genes in our mouse experiments. In experiments with human myogenic cells, DLL1 exhibits lower anti-myogenic functions compared to the other Delta-like protein DLL4, suggesting that DLL1 and DLL4 act differentially with Notch receptor. Although the molecular basis for this distinct function remains unresolved, it was demonstrated that Dll1 could trigger T cell lineage commitment via either Notch1 or Notch2, whereas Dll4 exclusively interacts with Notch1 [34]. In the present study, mRNA expression of *HES1* was similarly induced by all the three NOTCH ligands tested. However, *HEY1* and *HEYL* expression levels were not remarkably induced by DLL1 compared to DLL4 and JAG1 on human myoblasts. In studies with adult mouse myogenic cells, we reported previously that Hey1 and HeyL are essential for generating undifferentiated and quiescent muscle stem cells during postnatal development [17], suggesting that inefficient induction of *HEY1* and *HEYL* by DLL1 results in the insufficient *MyoD* suppression. This might also be the case for mouse Jag1. In human cells, the mechanism underlying the induction of *HEY1* and *HEYL* mRNA might explain the different effects of DLL1 and DLL4.

We present two unexpected data in the present study. First, Pax7 expression was increased in mouse cells while it was unchanged in human cells following treatment with Notch-ligand. The precise mechanism for this difference is unknown, but we previously reported that a Notch effector gene, *HES1* is strongly induced in human cells, but not mouse cells [26]. On the other hand, *HeyL* is significantly induced in mouse cells, but its induction in human cells is modest. Similarly, there might be species differences between human and mouse concerning *Pax7* expression. Second, mouse Jag1 did not strongly upregulate canonical Notch effectors genes including *Hes1* and *HeyL* in mouse myogenic cells, but it did suppress MyoD and Myogenin expression significantly at the protein level. Only *Hey1* was slightly upregulated in Jag1-Fc treated mouse cells. Previous work had shown that Hey1 inhibits skeletal muscle differentiation by reducing recruitment of MyoD to promoters [35], however, our study raises the possibility that Jag1 has anti-myogenic effect in a Hey1/L-independent manner in mouse myogenic cells.

In conclusion, Notch signaling promotes the expansion of Pax7+/MyoD- muscle stem-like cells and inhibits differentiation in vitro. We report that artificial maintenance of Notch signaling is not sufficient for retaining regenerative capacity of donor-cells after transplantation. The mechanism suppressing MYOD expression is crucial to improve myoblast engraftment potential since *MyoD* expression has a negative impact on murine myoblast survival [36]. It would be interesting in the future to explore additional pathways that can work in combination with Notch, or prolonged Notch signaling in specific contexts, to improve myoblast engraftment efficiency.

## Materials and methods

### Mice

Animals were handled according to national and European Community guidelines, and an ethics committee of the Institut Pasteur (CTEA) in France approved protocols. All procedures done in Japan for experimental animals were approved by the Experimental Animal Care and Use Committee at Osaka University. *Tg*:*Pax7-nGFP* [22] and *Dmd*^*mdx-βgeo*^ [23] mice were described previously. Immunodeficient *NOD-Scid* mice were purchased from Charles River (Yokohama, Kanagawa, Japan).

### Mouse cell isolation, culture, and induction of Notch signaling

Isolation of mouse muscle stem cells from *Tg*:*Pax7-nGFP* mice was performed as described previously [22, 37]. Briefly, mice were sacrificed by cervical dislocation, and muscles were chopped in cold DMEM and put into a 50-ml tube containing 30 ml of DMEM (Gibco, 31966), 0.1% Collagenase D (Roche, 1088866), 0.25% trypsin (Gibco, 15090–046), DNase 10 μg/ml (Roche, 11284932001) at 37°C under gentle agitation for 30 min. After standing still for 5 min at room temperature, the supernatants were collected into 5 ml fetal bovine serum (FBS, Gibco) on ice. The digestion was repeated for additional 4 times allowing complete digestion of the muscle. The supernatants were filtered through a 100-μm and then 70-μm cell strainer (BD Falcon). Cells were spun for 15 min at 600 RCF at 4°C, the pellets were resuspended in 1 ml of DMEM containing 2% FBS and filtered through a 40-μm cell strainer (BD Falcon) before cell sorting. Cells were isolated based on size, granulosity and GFP levels using a FACSAria II Cell Sorter (BD).

Jagged-1 fused to the Fc fragment of human IgG (Jag1-Fc) was purchased from AdipoGen International (AG-40A-0157T-C050). Human embryonic kidney (HEK) 293 cells were transfected with Delta-like 1-Fc (Dll1-Fc) [38] (kindly provided by N. Gupta upon permission from G. Weinmaster), Delta-like 4-Fc (Dll4-Fc) [21] (kindly provided by K. Alitalo and W. Zheng), or the Fc fragment only (Control-Fc) (kindly provided by T. Kadesch) to obtain stable cell lines. The conditioned medium was collected and concentrated with Centrifugal Filter Devices (10 kD, Millipore, UFC901024). Fc-fused proteins were purified by Protein G Sepharose (GE Healthcare, 17-0618-01). Protein concentration was determined by absorptiometer.

Culture dishes were first coated with 10 μg/ml of anti-Fc antibody (Jackson ImmunoResearch, 109-005-098) for 1h at room temperature. Dishes were coated with Matrigel (Corning, #354234) for 20 min, and then incubated with fusion proteins (5 ng/μl) for 1h at room temperature. Isolated mouse muscle stem cells were plated in culture media containing 20% FBS, 1% Penicillin-Streptomycin (Gibco, 15140), 2% Ultroser G (Pall Biosepra, 15950–017) in 50:50 DMEM:F12 (Gibco, 31966 and 31765) at 5000 cells per well of 6-well plate.

### Real-time PCR for mouse cells

Total RNA was extracted from the mouse cells by RNeasy Micro Kit (Qiagen, 74004). cDNA was prepared by random-primed reverse transcription (SuperScript II, Invitrogen, 18064–014). Real-time PCR was done using SYBR Green Universal Mix (Roche, 13608700) and StepOnePlus Real-Time PCR System (Applied Biosystems). Primer sequences are listed in supplementary material S1 Table.

### Immunocytochemistry for mouse cells

Cells were fixed with 4% paraformaldehyde (PFA) in cold PBS for 5 min, permeabilized with 0.5% Triton X-100 for 5 min, washed, and blocked with 10% BSA for 1h. Cells were incubated with primary antibodies overnight at 4°C. Antibodies used are listed in S2 Table. Cells were washed with PBS and then incubated for 1 h with secondary antibodies and Hoechst (1:10,000, Molecular Probes). Images were acquired using a Zeiss Axio-plan equipped with an Apotome and ZEN software (Carl Zeiss) or a confocal Leica Spe microscope (Leica).

### Transplantation of mouse cells, tissue processing, and immunohistochemistry

Mice were anesthetised by intraperitoneal injection of a solution of 0.9% NaCl_2_, 0.5% Imalgene (Merial), 2% Rompun (Bayer; 100 μl per 25 g of the mice weight). The TA muscles of *Dmd*^*mdx-βgeo*^ mice were injected with 50 μl of cardiotoxin from Naja pallida (10 μM, Latoxan, Catalog number; L8102, Rosans, France) the day before transplantation. For engraftments, 2 x 10^4^ cells in 15 μl of PBS were injected into TA muscles of anesthetized host mice. TA muscles were removed two weeks after transplantation. Injected muscles were frozen in liquid nitrogen-chilled isopentane.

For immunofluorescence staining, serial 10 μm cryosections were collected and blocked with 2% goat serum in PBS. Antibodies were diluted in blocking buffer. Antibodies used are listed in S2 Table. Hoechst-33342 was used to counter-stain nuclei. Images were acquired with a confocal Leica Spe microscope (Leica). For quantification of dystrophin+ fibers, serial transverse sections were cut throughout the entire TA muscle. Each TA muscle generated 20–25 slides, each slide consisting of 20–25 serial sections. Five different slides were stained for dystrophin. The one section in the slide with the maximum number of dystrophin+ myofibers for each animal was counted.

### Human myogenic cell isolation, culture, and induction of Notch signaling

Experiments using human samples were approved by the Ethical Review Board for Clinical Studies at Fujita Health University and Osaka University. Muscle samples were obtained from gluteus medius muscles of patients undergoing total hip arthroplasty as described before [24, 39]. Isolation of myogenic cells was done at Fujita Health University, and the frozen cells were used in Osaka University for following studies. All patients or their parents gave written informed consent.

Human myoblasts were isolated as described previously [24]. In brief, muscles were digested using 0.2% type II collagenase, passed through an 18 G needle, and isolated cells were cultured in DMEM-HG 20% FBS containing bFGF. After several days, cultured cells were trypsinized, resuspended and stained with anti-CD56 (NCAM-1) antibodies. After staining with streptavidin-PE/Cy5, CD56+ cells were sorted by FACSVantage SE (BD) and cultured in DMEM-HG 20% FBS containing bFGF.

All NOTCH ligands were purchased from AdipoGen International (San Diego, CA, USA). The catalog number of human Jagged-1 (JAG1) -Fc (Fc portion of human IgG1) chimera, Delta-like Protein 4 (DLL4) -Fc (Fc portion of human IgG1) chimera, or Delta-like Protein 1 (DLL1) -Fc (Fc portion of human IgG1) chimera are AG-40A-0081, AG-40A-0077Y, or AG-40A-0116Y, respectively.

When human myoblasts were stimulated by NOTCH ligand, culture plates were coated with NOTCH ligand in PBS at 5 ng/μl to 20 ng/μl for 150 min at 37°C CO_2_ incubator. After removing NOTCH ligand solution, cells were seeded in appropriate concentration and cultured at CO_2_ incubator for 24 h.

### Real-time PCR for human cells

Total RNA was extracted from the cells cultured with immobilized NOTCH ligands and control cells using RNeasy Micro Kit according to the manufacturer’s instructions (Qiagen, Hilden, Germany) and the reverse-transcribed into cDNA using TaqMan reverse transcription reagents (Roche Diagnostics, Mannheim, Germany). Primer sequences are listed in supplementary material S1 Table.

### Immunocytochemistry for human cells

Cultured human myoblasts were fixed with 4% PFA for 10 min and then permeabilized with 0.1% Triton-X-PBS for 20 min. After blocking with 5% skim milk, the cells were stained with primary antibodies. After the first staining at 4°C overnight, sections were incubated with secondary antibodies conjugated with Alexa Fluor 488, or 546 (Molecular Probes), Cy3 (Jackson ImmunoResearch Inc., West Grove, PA, USA). The detailed information of primary antibodies is listed in supplementary material S2 Table.

### Transplantation of human cells, tissue processing, and immunohistochemistry

Anesthesia was induced with sevoflurane (Wako). One day prior to cell engraftment, 100 μl of cardiotoxin from *Naja mossambica mossambica* (10 μM in saline, Sigma-Aldrich, Catalog number; C9759-5MG St. Louis, MO, USA) was injected into the TA muscles in order to induce regeneration. The myoblasts cultured with or without JAG1 or DLL4 were harvested, resuspended in 30 μl PBS and then transplanted into the injured TA muscles. Two weeks after transplantation, mice were euthanized by cervical dislocation, and then the TA muscles were excised and rapidly frozen in liquid nitrogen-cooled isopentane (Wako Pure Chemical Industries, Osaka, Japan). The transplanted muscles were sectioned at 250 μm intervals from the proximal end to the mid-belly.

For LAMIN AC-staining, transverse cryosections (6 μm) were fixed with 4% PFA for 10 min. For SPECTRIN-staining, the sections were fixed with cooled acetone for 10 min at -20°C. After blocking with an M.O.M. Kit (Vector Laboratories, Burlingame, CA, USA) in 5% skimmed milk, sections were stained with primary antibodies. After the first staining at 4°C overnight, sections were incubated with secondary antibodies conjugated with Alexa Fluor 488, or 546 (Molecular Probes, Eugene, OR, USA). The maximal number of human SPECTRIN+ myofibers was recorded. Detailed information of primary antibodies is listed in supplementary material S2 Table.

### Statistics

Statistical significance was assessed by Welch’s t-test followed by the Bonferroni test, 2-sample test for equality of proportions with Holm method, or non-repeated measures analysis of variance (ANOVA) followed by the Bonferroni test. A probability of less than 5% (p < 0.05) or 1% (p < 0.01) was considered statistically significant.

## Supporting information

S1 Table Primer sequences for real-time PCR. (PDF)

S2 Table List of primary antibodies. (PDF)
