# Supplementary material for: Notch ligands regulate the muscle stem-like state ex vivo but are not sufficient for retaining regenerative capacity
Source: PLoS One. 2017 May 12;12(5):e0177516. doi: 10.1371/journal.pone.0177516 (PMC5428926; doi:10.1371/journal.pone.0177516)
Supplement: S1 Table — (PDF) [file pone.0177516.s001.pdf]

S1 Table. Primer sequences for Real-time PCR.

For mouse Exp.		Sequence	Product (bp)
Hes1	Fwd	ACACCGGACAAACCAAAGAC	148
	Rev	AATGCCGGGAGCTATCTTTC	
Hey1	Fwd	CACCTGAAAATGCTGCACAC	122
	Rev	ATGCTCAGATAACGGGCAAC	
HeyL	Fwd	GTCTTGCAGATGACCGTGGA	73
	Rev	CTCGGGCATCAAAGAACCCT	
MyoD	Fwd	CACTACAGTGGCGACTCAGATGCA	144
	Rev	CCTGGACTCGCGCGCCGCCTCACT	
Pax7	Fwd	GACAAAGGGAACCGTCTGGAT	96
	Rev	TGTGAACGTGGTCCGACTG	
Rpl13a	Fwd	GTGGTCCCTGCTGCTCTCAAG	152
	Rev	CGATAGTGCATCTTGGCCTTTT	
**For human Exp.**		**Sequence**	**Product (bp)**
HES1	Fwd	AAGAAAGATAGCTCGCGGCA	134
	Rev	TACTTCCCCAGCACACTTGG	
HEY1	Fwd	CCTTCCCCTTCTCTTTCGGC	126
	Rev	AAAAGCTCCGATCTCCGTCC	
HEYL	Fwd	AGACCGCATCAACAGTAGCC	115
	Rev	GTGATCCACCGTCATCTGC	
MYOD1	Fwd	GCCACAACGGACGACTTCTATG	116
	Rev	TGCTCTTCGGGTTTCAGGAG	
PAX7	Fwd	GACCCCTGCCTAACCACATC	133
	Rev	GTCTCCTGGTAGCGGCAAAG	
GAPDH	Fwd	CCATCACCATCTTCCAGGAG	117
	Rev	AATGAGCCCCAGCCTTCTCC	
