# Supplementary material for: Notch ligands regulate the muscle stem-like state ex vivo but are not sufficient for retaining regenerative capacity
Source: PLoS One. 2017 May 12;12(5):e0177516. doi: 10.1371/journal.pone.0177516 (PMC5428926; doi:10.1371/journal.pone.0177516)
Supplement: S2 Table — (PDF) [file pone.0177516.s002.pdf]

S2 Table. List of primary antibodies.

Antibody	Clone	Ig type	Supplier	Catalog number	Application and dilution
**For mouse Exp.**					
Pax7	PAX7	Mouse IgG1, k	DSHB	PAX7	ICC: x20 (supernatant)
MyoD	5.8A	Mouse IgG1, k	DAKO	M3512	ICC: x200
Myogenin	F5D	Mouse IgG1, k	DSHB	F5D	ICC: x20
Dystrophin	Polyclonal	Rabbit IgG	Abcam	ab15277	IHC: x500
**For human Exp.**					
PAX7	PAX7	Mouse IgG1, k	DSHB	Pax7	ICC: x2 (supernatant)
MYOD	EPR6653-131	Rabbit IgG	Abcam	ab133627	ICC: x150
SPECTRIN	RBC2/3D5	Mouse IgG2b	Abnova	MAB9510	IHC: x100
LAMIN A/C	EPR4100	Rabbit IgG	Abcam	ab108595	IHC: x400
